# The angular gyrus serves as an interface between the non-lexical reading network and the semantic system: evidence from dynamic causal modeling

**DOI:** 10.1007/s00429-023-02624-z

**Published:** 2023-03-11

**Authors:** Frederick Benjamin Junker, Lara Schlaffke, Joachim Lange, Tobias Schmidt-Wilcke

**Affiliations:** 1 Department of Neuropsychology, Faculty of Psychology, Ruhr-University Bochum, Universitätsstraße 150, 44801 Bochum, Germany; 2 Institute of Clinical Neuroscience and Medical Psychology, Medical Faculty, Heinrich Heine University, Universitätsstraße 1, 40225 Düsseldorf, Germany; 3 Department for Neurology, Professional Association Berufsgenossenschaft-University Hospital Bergmannsheil, Bürkle de La Camp-Platz 1, 44789 Bochum, Germany; 4 Neurological Center Mainkofen, Mainkofen A 3, 94469 Deggendorf, Germany

**Keywords:** Dynamic causal modeling, Reading model, Non-lexical processing, Phonological lexicon, Semantic system, Angular gyrus

## Abstract

Understanding encoded language, such as written words, requires multiple cognitive processes that act in a parallel and interactive fashion. These processes and their interactions, however, are not fully understood. Various conceptual and methodical approaches including computational modeling and neuroimaging have been applied to better understand the neural underpinnings of these complex processes in the human brain. In this study, we tested different predictions of cortical interactions that derived from computational models for reading using dynamic causal modeling. Morse code was used as a model for non-lexical decoding followed by a lexical-decision during a functional magnetic resonance examination. Our results suggest that individual letters are first converted into phonemes within the left supramarginal gyrus, followed by a phoneme assembly to reconstruct word phonology, involving the left inferior frontal cortex. To allow the identification and comprehension of known words, the inferior frontal cortex then interacts with the semantic system via the left angular gyrus. As such, the left angular gyrus is likely to host phonological and semantic representations and serves as a bidirectional interface between the networks involved in language perception and word comprehension.

**Supplementary Information:**

The online version contains supplementary material available at 10.1007/s00429-023-02624-z.

## Introduction

Translating phonological speech into written orthography and vice versa are highly demanding cognitive skills that have significantly contributed to the evolution of human culture. In alphabetic languages, encoding is achieved by combining graphemes to represent the phonology of individual words. Decoding alphabetic languages is thought to rely on two separate processes dependent on the orthographic regularity of a word. On the one hand, regular words can be—and unknown words must be—decoded in a letter-by-letter fashion (non-lexical decoding). This decoding utilizes regularities in the grapheme-phoneme correspondence (GPC; Protopapas et al. 2016; Yap et al. 2015), where the phonology of a word can be reconstructed based on the individual phonemes. On the other hand, irregular words that violate these regularities need to be decoded involving item-specific knowledge, which is possible once the word has become familiar to the reader (lexical decoding; e.g., Graves et al. 2010). Reading models addressing both decoding strategies have been strongly influenced by the investigation of patients with acquired dyslexia, who suffer from impaired reading performance of either regular (phonological dyslexia) or irregular words (surface dyslexia). Both, phonological and surface dyslexia can be associated with lesions in distinct brain regions (Tomasino et al. 2020), indicating that the cognitive processes involved in non-lexical and lexical decoding rely on at least partially distinct brain regions. At the same time, decoding in experienced readers, such as adults, is likely to involve both non-lexical and lexical decoding strategies simultaneously in an interactive manner (Barton et al. 2014). Various approaches such as computational modeling have been used to explain the interaction of non-lexical and lexical decoding in healthy subjects (Levy et al. 2009) and dyslexic patients (Ziegler et al. 2008; Bergmann and Wimmer 2008; for a review, see Rapcsak et al. 2007).

**Fig. 1:**
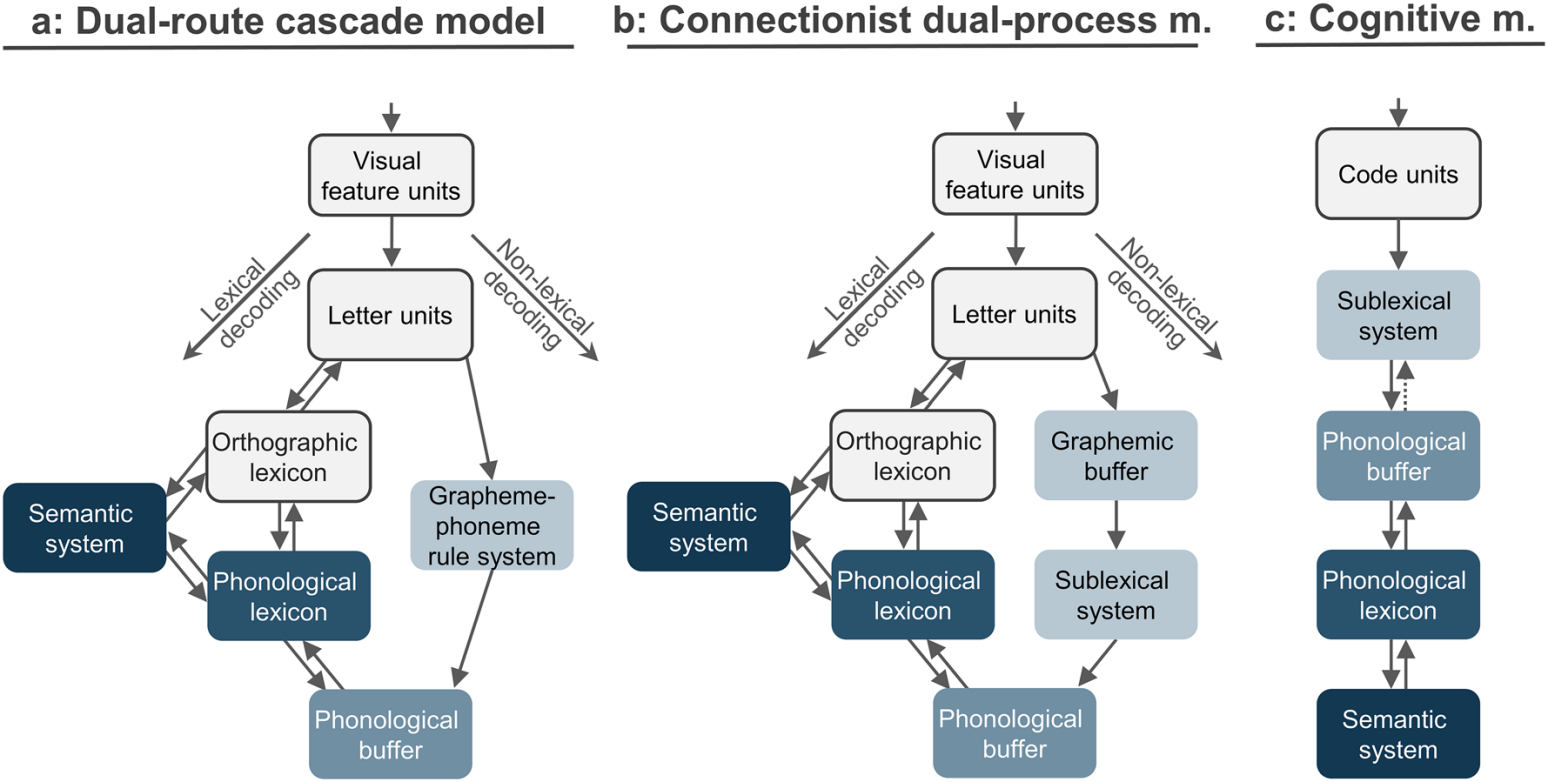
Models for language decoding. Dual-route cascade (DRC; **a**) and connectionist dual-process (CDP; **b**) model for reading. Both models were joined into a cognitive model for non-lexical decoding used for later modeling (**c**). This model includes possible bidirectional connections from the phonological buffer to the sublexical system (top-down: dotted line) that is not included in DRC or DCP

Common computational models like the dual-route cascade (DRC; Coltheart et al. 2001) and the connectionist dual-process (CDP; Perry et al. 2007) model use two parallel decoding routes to simulate the interaction of non-lexical and lexical decoding (see Fig. 1). The lexical route links the orthography of known words to their meaning (semantic system) either directly or indirectly via their phonology (phonological lexicon) and is therefore crucial for decoding irregular words (Coltheart et al. 2001). In contrast, the non-lexical route reconstructs the phonology of the word via a serial conversion of the individual graphemes (sublexical system) followed by a subsequent storage and assembly of the resolved phonemes (phonological buffer). As outlined above, this route is essential for reading unfamiliar words or artificial nonwords. Although the DRC and CDP models share an identical lexical route, they differ in their implementation of the non-lexical route. While the DRC model assumes a rule-based grapheme-phoneme network (see Fig. 1a), the CDP model uses a parallel two-layered association network to extract the statistically most reliable GPC (sublexical system) with a preceding graphemic buffer (see Fig. 1b). In both models, associations between the phonology (phonological lexicon) and semantics (semantic system) enable subsequent comprehension of known words. Although both models differ in their computational implementation, they can be criticized for their pure bottom-up view on non-lexical decoding.

This unidirectional processing alone cannot account for the ‘word superiority effect’, in which the recognition of individual letters can be enhanced when written in the context of a word or readable nonword (Ripamonti et al. 2018). Accordingly, additional modulation via top-down connections might be required, suggesting a bidirectional network for non-lexical decoding. Furthermore, neither DRC nor CDP include a computational implementation of semantics, representing only a framework for word comprehension (Seidenberg 2012). Nevertheless, both models reflect the main processes involved in understanding encoded language, yielding a priori hypotheses for the cognitive network architecture involved in non-lexical language decoding (see Fig. 1c). In this way, these models represent a suitable framework to guide the interpretation of brain imaging data (e.g., Taylor et al. 2013). Importantly, the DRC and CDP are computational models that per se do not assign the postulated functions to any specific brain regions. However, due to the high reading speed and parallel lexical and non-lexical decoding, the mapping of these functions remains challenging.

Although the usage of artificial nonwords and irregular words allows at least a partial distinction between non-lexical and lexical decoding strategies, a parallel activation of the two decoding routes cannot be ruled out (Levy et al. 2009). Both routes are not fully independent and interact at both the phonological and semantic level (Rapcsak et al. 2007). To circumvent these challenges, we previously used the international Morse code (MC) as a model for language learning and decoding, allowing us to probe exclusively the non-lexical decoding strategy followed by a lexical-decision (for a detailed description, see Junker et al. 2020). While MC and written script differ perceptually (auditory vs. visual) and in their way of encoding (temporal vs. spatial encoding), both require the same (or at least similar) cognitive computations to be decoded and understood. During non-lexical decoding, individual graphemes must be translated and combined. For comprehension, the phonology of a known word must then be reconstructed, leading to a (re)activation of semantic associations learned during language acquisition. These decoding processes are unique to encoded languages such as written script or MC and are not required for speech perception. In contrast to verbal language, which has evolved at least within the modern Homo sapiens over the past 200.000 years (Richter et al. 2017), reading and writing are a fairly recent invention (first evidence for alphabetic languages around 2000 BC (Darnell et al. 2005)). As no cortical specialization for reading can be assumed within this short period (Tooby and Cosmides 2000), language decoding makes use of pre-existing cognitive features (Dehaene et al. 2010, 2015). Since the cognitive computations (and their underlying neuronal resources) for non-lexical decoding of written script and MC are the same, MC can be used as a (limited) model for language learning and decoding.

Using MC and fMRI, we previously identified brain activations associated with non-lexical decoding (sublexical system and phonological buffer) in the left inferior parietal lobule and inferior frontal cortex (non-lexical decoding network; Junker et al. 2020). Additional activations associated with word comprehension (lexicality effect) were found in the left angular gyrus, the anterior cingulate cortex, and the precuneus, indicating subsequent lexical and semantic processing (comprehension network). Accordingly, the brain regions that host the cognitive computations required for decoding and comprehension of MC and written script (e.g., Taylor et al. 2013) are highly consistent, further supporting the concept of feature-specific rather than modality-specific representations. However, this study used a univariate data analysis within the framework of the general linear model (GLM) to identify brain regions associated with non-lexical decoding and word comprehension. As this analysis is not capable to detect cross-regional interactions (as expected during language decoding), further classifications were not possible in this prior study.

To investigate causal interactions across brain regions using functional brain imaging, advanced analysis techniques such as dynamic causal modeling (DCM) are required (Friston 2009). In contrast to alternative methods for effective connectivity analyses, DCM combines a neurobiological model for neural dynamics and a biophysical model to describe the transformation of neural activity to the measured BOLD signal, minimizing the influence of regional differences in hemodynamic response (Friston et al. 2013). DCM uses Bayesian model selection to identify significant families of models as well as the most likely individual model architecture, which takes both model performance and complexity into account (Stephan and Friston 2010). In addition, DCM aims to estimate various model parameters, describing how activity in an area is affected by intrinsic and latent static connections (A matrix), as well as modulatory experimental influences (C matrix). Additionally, the direct influence of experimental influences on the effective connectivity between regions can be estimated (B matrix).

In this study, we further investigated the non-lexical route using fMRI and DCM, with a specific interest in the interaction of brain regions involved in non-lexical decoding (sublexical system, phonological buffer) and word comprehension (phonological lexicon, semantic system). Based on the brain regions identified by task-related fMRI (while decoding MC) and applying the cognitive model for non-lexical language decoding (see Fig. 1c), we sought to disentangle the functional network architecture with different subcomponents enabling decoding and comprehension. More specifically, we investigated the interaction of two intertwined networks subserving non-lexical decoding and word comprehension; i.e., the translation, short-term storage and assembly of phonological units (phonemes), and the subsequent identification of a (known) word. We also sought to identify the connecting hubs, enabling an information flow between the decoding and comprehension network.

## Material and methods

### Subjects

Thirty-three participants (18 male) between 18 and 30 years (mean: 23; standard deviation: 2) took part in the experiment. All participants were naïve to Morse code (MC). Exclusion criteria included metal implants (retainers, pacemakers, etc.), neurological or psychiatric history, and claustrophobia. The handedness was restricted to right-handed persons and controlled using the Edinburgh handedness inventory (Oldfield 1971). All participants gave written informed consent before the study was performed.

### Training

All participants learned 12 Morse code (MC) letters in 6 separate lessons spaced across a maximum time range of 12 days using an audiobook. These letters (A, D, E, G, I, M, N, O, R, S, T, and U) were chosen so that their MC consisted of a maximum of three signals (short or long) and that a sufficient number of German words could be formed. Each audiobook lesson consisted of 5–10 blocks (30–60 letters per block) and was completed on-site in the laboratory. If subjects failed to reach a certain learning target per lesson (e.g., errors in three of the last six blocks), a repetition of the last block was conducted (~ 5 min). Although the learning procedure was similar for all subjects, it differed in the sensory modalities involved. While some subjects learned the MC as purely auditory sequences (Junker et al. 2021: Unisensory learners; 17 subjects), others additionally perceived the MC as vibrotactile sequences applied to the left hand (Junker et al. 2021: Multisensory low-level learners; 16 subjects). However, since training-related differences between these groups were only found in right-hemispheric brain regions associated with tactile perception (postcentral gyrus) and multisensory integration (inferior frontal cortex), both groups were analyzed together in the present study. Importantly, all subjects spent the same amount of time exercising (for a detailed description of the learning procedure, see Junker et al. 2021).

### Task

#### Stimuli

A total of 40 German words (mean duration: 3.57 s; e.g., ‘RAD’ or ‘NOT’) and 40 meaningless nonwords (mean duration: 3.56 s; e.g., ‘ENS’ or ‘RUO’) were used in the experiment and presented as auditory MC (for a detailed list of all stimuli, see Supplementary Table 1). Only three-letter stimuli were used, as these provided a good balance between possible words (*n* = 86) and working memory load. The words included 37 nouns and 3 adjectives, while the nonwords included 30 pronounceable pseudowords and 10 unpronounceable nonwords. All words and nonwords included 79 bigrams and 11 multi-letter graphemes. The average word frequency was 14.7 instances per million words (standard deviation = 43.98) and was measured based on a German word corpus comprising the literature between 2000 and 2010 (DWDS core corpus 21; including over 121 million words from Fiction, Popular Literature, Science, and Journalistic Prose). In addition, the international SOS signal in MC (mean duration: 2.28 s) as well as a 796 Hz sinusoidal tone (mean duration: 3.7 s) were presented 25 times each and served as control stimuli (only sinusoidal tone analyzed here).

#### Lexical-decision task

Subjects performed a lexical-decision task before and after learning while we simultaneously recorded neural activity using functional magnetic resonance imaging (fMRI). For this purpose, words and nonwords were presented as auditory MC sequences. Subjects had to decide whether the presented letters represent a German word or a nonword. Additionally, both control tones were presented and had to be identified. All stimuli were presented in randomized order using the software Presentation^®^ (Neurobehavioral Systems, Albany, CA, USA). The subjects communicated their answers by pressing a keypad with the left pinky (word), ring (nonword), middle (SOS signal), or index finger (control). In addition to the lexical-decision task, the subjects performed a perceptual task using the same stimulus material, which has been described elsewhere (see Junker et al. 2021). Each task was divided into two sessions, allowing for a short break between the sessions. However, only the data from the lexical-decision task after training were analyzed here, as the subjects could only decode the MC stimuli after training (for a comparison of the data before and after learning, see Schlaffke et al. 2015).

#### Behavior

Statistical analysis of the behavioral data was performed using IBM SPSS (version 20), aiming to investigate stimulus-specific differences in recognition performance and response times (stimuli comparisons). To test for statistical differences, the normality of the data was tested using the Shapiro–Wilk test before the stimuli were compared using either parametric analysis of variance or non-parametric Kruskal–Wallis test. Post hoc pairwise comparisons were performed using either student’s *t*-tests or Mann–Whitney-*U*-test for normal or non-normal distributed data, respectively. Bonferroni correction for multiple comparisons was performed across stimuli comparisons (recognition performance and response times; *n* = 2) and corresponding post hoc tests (*n* = 3) by multiplying the calculated p-values by the number of tests performed. This correction was chosen since an interpretable alpha error of 5% will be maintained. However, as this correction procedure can yield p-values above 1, high *p*-values are indicated as “ > 1”.

### Magnetic resonance imaging

#### Acquisition

To investigate the neural basis of MC decoding, structural and functional magnetic resonance (MR) images were acquired at the University Hospital Bergmannsheil in Bochum (Germany), using a 3 T MR scanner and a 32-channel head coil (Philips Achieva 3.2, Best, Netherlands). Auditory stimuli were presented via MR-compatible headphones. Furthermore, protection was provided against the scanner noises. All stimuli were presented at a volume that could be easily heard by the subject. In addition, MR-compatible LCD goggles were used, via which the task instruction and response options were displayed during the experiment.

After preparation of the subject, a structural high-resolved T1 weighted image was acquired (~ 5 min), resulting in an isometric resolution of 1 mm (field of view: 256 × 256 × 220 mm^3^; repetition times TR: 8.3 ms; echo times TE: 3.8 ms). Subsequently, T2*-weighted echo-planar images were acquired while the subjects performed a perceptual (not investigated here) and lexical-decision task (TR: 2400 ms; TE: 35 ms; flip-angle: 90°). 250 dynamic scans were recorded per session (2x ~ 10 min), while no stimulus was presented during the first four (dummy) scans. Each scan consisted of 36 slices measured in ascending order, resulting in a voxel size of 2 × 2 × 3 mm^3^ (field of view of 256 × 256 × 108 mm^3^).

#### Preprocessing

The analysis of the MR data was performed using the software SPM12 (Statistical Parametric Mapping, Welcome Department of Cognitive Neurology, University College, London, UK) running under Matlab 2019a (The MathWorks Inc., Massachusetts, USA). After the removal of the first four images, which served as dummy scans, temporal correction of the consecutively acquired slices was first performed (slice-time correction). Subsequently, the 246 dynamic recordings were realigned by back-rotation and back-translation to correct for subject motion during recording. One session was excluded from the further analysis in one subject due to strong head movements during data acquisition (> 5 mm in *x*, *y*, or *z*). To transform the MR images into the normalized MNI space, the functional images were first coregistered to the high-resolution structural image. Subsequently, the structural images were decomposed into gray matter, white matter, cerebrospinal fluid, bone, and soft tissue (segmentation). The deformation field calculated during the segregation was then used to normalize the functional (and coregistered) data. Finally, the functional images were spatially smoothed using a Gaussian kernel (full width at half maximum: 6 × 6 × 6 mm^3^) to normalize the error distribution, improve the signal-to-noise ratio and adjust for inter-individual variations.

#### General linear model

To investigate the effective connectivity of brain regions during non-lexical MC decoding, the brain regions that are critically involved in processing must be identified first. To identify these core regions, a univariate analysis was performed in the statistical framework of a general linear model implemented in SPM12. The resulting clusters were subsequently labeled using the Automated Anatomical Labelling III atlas (https://www.gin.cnrs.fr/en/tools/aal/) and visualized using the SPM toolboxes bspmview (http://www.bobspunt.com/bspm-view/).

During the first-level analysis, the individual events were first modeled as box-car functions and convolved with the hemodynamic response to find a model that best explained the data. As events, the correctly identified stimulus types (words, nonwords, control) were modulated separately between stimulus offset and the subject's response. If subjects responded before the stimulus offset (possible only for control tones), a stick function was used for modeling (instead of a box-car function). The time window between offset and response was chosen, as this period allows the identification of core brain regions related to both, language decoding and word comprehension (for details, see Junker et al. 2020). However, to keep the analysis simple, no additional time window was analyzed here. Nevertheless, the period while the stimuli were presented (onset-offset), the SOS signal, as well as all unidentified stimuli were modeled to exclude an effect on the implicit baseline. Furthermore, the individual rotation and translation parameters calculated during preprocessing (realignment, see “Preprocessing”) were used as additional covariates of no interest. After estimating the model, the individual conditions (words, nonwords, control) were contrasted against the implicit baseline for the subsequent second-level model.

Second-level group analyses were performed using correctly identified words, nonwords, and control tones only. To identify brain regions involved in the conversion of MC letters (sublexical system) and the subsequent storage and assembly of the resulting phonemes (phonological buffer), words and nonwords were compared against the control tone using a conjunction contrast ([words > control] ^ [nonwords > control]). Although the retrieval of semantic meaning was not crucial to perform the lexical-decision task, the data were analyzed and discussed in the context of semantic processing, since reaction times during lexical decisions are influenced by semantics. For example, lexical processing speeds are influenced by various semantic richness measures such as the number of semantic neighbors, the number of semantic features, or contextual dispersion (Pexman et al. 2008). This influence of semantic properties on processing speeds suggests that semantic representations are involved in lexical processing and are retrieved automatically (Balota 1983), although semantic retrieval was not required to perform the lexical-decision task. Therefore, words were contrasted against nonwords to identify brain regions involved in representing phonological and semantic features of known stimuli (words > nonwords; lexicality effect). Whole-brain analysis was performed with an initial significance level of *p*_Voxel_ < 0.001, corrected for multiple comparisons at the cluster-level (family-wise error correction, *p*_Cluster_ < 0.05).

From these results, five different left-hemispheric peak activations were selected. Subsequently, a sphere (radius: 6 mm) centered on the selected peak voxel was created, serving as ROIs for the effective connectivity analysis. The opercular part of the inferior frontal cortex (IFC—ROI 1) as well as the supramarginal gyrus (SMG—ROI 2) were engaged during word and nonword decoding, suggesting an involvement in the sublexical system or phonological buffer. We especially selected peak activations within the IFC and inferior parietal lobule (SMG), as these regions are most commonly described in the literature for non-lexical language decoding (see “Discussion”). In contrast, the angular gyrus (AG—ROI 3), as well as the anterior cingulate cortex (ACC—ROI 4) and precuneus (PC—ROI 5) were exhibited while decoding words only, suggesting a participation in representing phonological (phonological lexicon) and semantic (semantic system) features on known stimuli. Although only two cognitive components were expected, all three areas were nevertheless used for subsequent modeling, since the AG, ACC, and PC are likely to represent one functional network (default mode network). If multiple peak activations were found within one region (as found within the ACC, AG, and IFC), the peak with the highest statistical *z*-value was selected. Although additional regions were also involved in non-lexical decoding (e.g., insular cortex) and word comprehension (e.g., posterior middle temporal cortex), we restricted the subsequent effective connectivity analysis to five regions to keep the models simple (for more details, see “Limitations”).

#### Dynamic causal modeling

Effective connectivity analyses were performed using DCM implemented in SPM12 (version 12). DCM aims to estimate various model parameters, describing how activity in an area is affected by intrinsic and latent static connections (A matrix) as well as modulatory experimental influences (C matrix). Additionally, the direct influence of experimental influences on the effective connectivity between regions can be estimated (B matrix). Bilinear DCM was applied on mean-centered data using one state per region (inhibitory and excitatory effects combined) and no stochastic effects (e.g., state-dependent processes such as short-term plasticity). All models were first created for each subject before the connectivity strengths (A, B, and C matrix) and their probabilities were estimated.

To identify the most likely individual model architecture across all subjects, Bayesian Model Selection was performed assuming fixed effects, taking into account both model performance and complexity (Stephan and Friston 2010). In contrast to random effects, where various cognitive strategies could be assumed to perform the same task (e.g., lexical and non-lexical decoding), fixed effects were expected since only non-lexical decoding strategies could be applied after learning single letters in MC. Therefore, differences in log-evidence (difference in log-evidence to worst model) are reported, where a difference of three can be interpreted as strong evidence (Stephan and Friston 2010). In addition to the individual model comparison, Bayesian Model Selection was applied across groups of models based on their feature membership (families) to compare specific hypotheses against each other. For model families, differences in summed log-evidence (sum across all models of one family) compared to the worst family are reported. However, no Bayesian model averaging was performed across families of similar features, since we were interested in the most likely model and the corresponding brain mapping. Finally, all connectivity parameters (A, B, and C matrix) as well as their probabilities of the most likely model were averaged across subjects using Bayesian parameter averaging.

As described above, only a reduced time window had been investigated in the GLM (stimulus offset to button press), since this processing phase allows the investigation of brain activations related to language decoding and comprehension (for detailed information, see Junker et al. 2020). However, since we cannot determine exactly when the comprehension network becomes involved in processing, we modeled the entire processing time window (stimulus onset to button press) for the subsequent DCM analysis. In this way, we could ensure to capture both, the early initialization of MC decoding and the later transition to word comprehension, allowing us to test specific hypotheses and models.

**Fig. 2:**
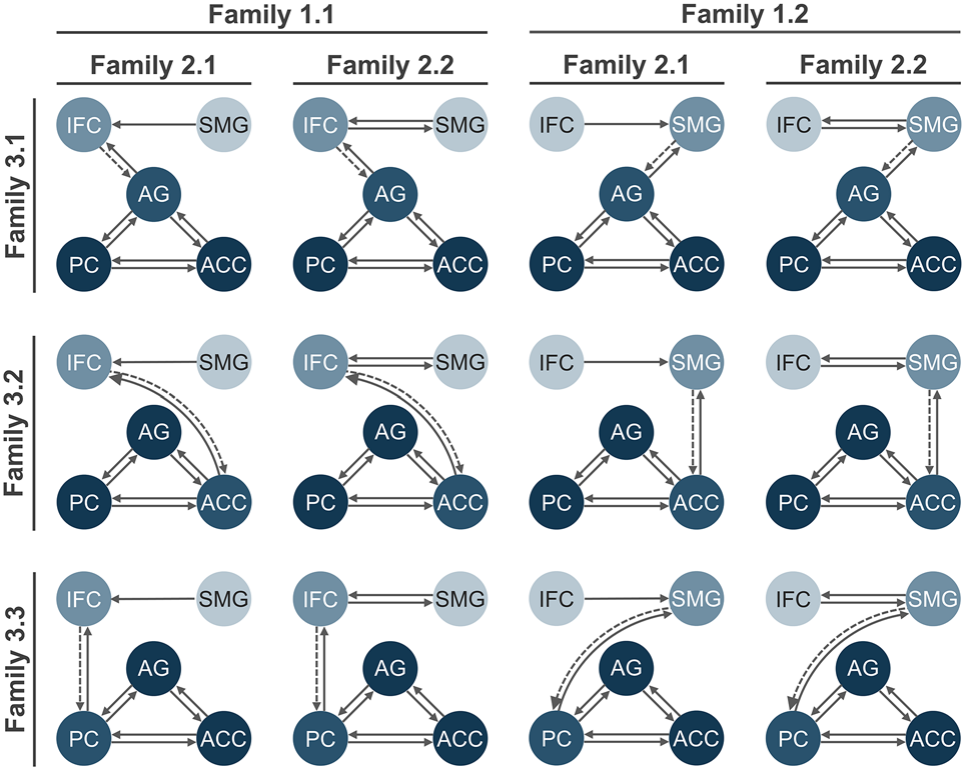
DCM models. All 12 DCM models tested, sorted by family. Words and nonwords served as modulatory input to the sublexical system (light blue; C matrix), located either in the SMG (Family 1.1) or IFC (Family 1.2). The sublexical system connects to the phonological buffer (medium blue; **A** matrix) involving unidirectional (Family 2.1) or bidirectional connections (Family 2.2). Words additionally influence the effective connectivity (dashed line; **B** matrix) between the phonological buffer and phonological lexicon (blue) within the AG (Family 3.1), ACC (Family 3.2), or PC (Family 3.3). The phonological lexicon interacts with brain regions involved in the semantic system (dark blue)

The models tested here arose from the observations in dyslexic patients as well as from computational models of language decoding and criticisms thereof, e.g., lack of top-down connections. The following assumptions were made: (1) Different processes involved in language decoding are represented in distinct brain regions, as suggested by neuroimaging of healthy subjects and dyslexic patients. (2) The brain regions responsible for sublexical conversions (sublexical system) and phonological working memory (buffer) are involved in decoding words and nonwords. (3) These regions are serially connected (one after the other), involving either unidirectional (bottom-up) or bidirectional (bottom-up and top-down) connections (criticisms of computational models). (4) Words additionally modify the connectivity from the phonological buffer to the phonological lexicon, which subsequently interacts with the semantic system to enable word comprehension (see Fig. 1c). Based on these assumptions, 12 different models were tested, which can be divided into three feature families (see Fig. 2).

Family 1: Words and nonwords serving as modulatory input to the non-lexical decoding network (C matrix) via the SMG (Family 1.1—6 models) or IFC (Family 1.2—6 models). While Family 1.1 reflects an involvement of the SMG in the sublexical system and the IFC in the phonological buffer, Family 1.2 represents the opposite hypothesis.

Family 2: The sublexical system and phonological buffers communicate via unidirectional (Family 2.1—6 models) or bidirectional connections (Family 2.2—6 models; A matrix). As suggested by the ‘word superiority effect’ in nonwords, non-lexical decoding might involve additional top-down connections, which are not included in the DRC or CDP model. This hypothesis is represented by Family 2.2, while Family 2.1 reflects pure unidirectional bottom-up connections within the decoding network.

Family 3: Words additionally modulate the connection from the phonological buffers to the AG (Family 3.1—4 models), ACC (Family 3.2—4 models), or PC (Family 3.3—4 models; B matrix). Each subfamily represents the hypothesis that the phonological lexicon is localized in either the AG, ACC, or PC, respectively.

## Results

### Behavior

**Fig. 3:**
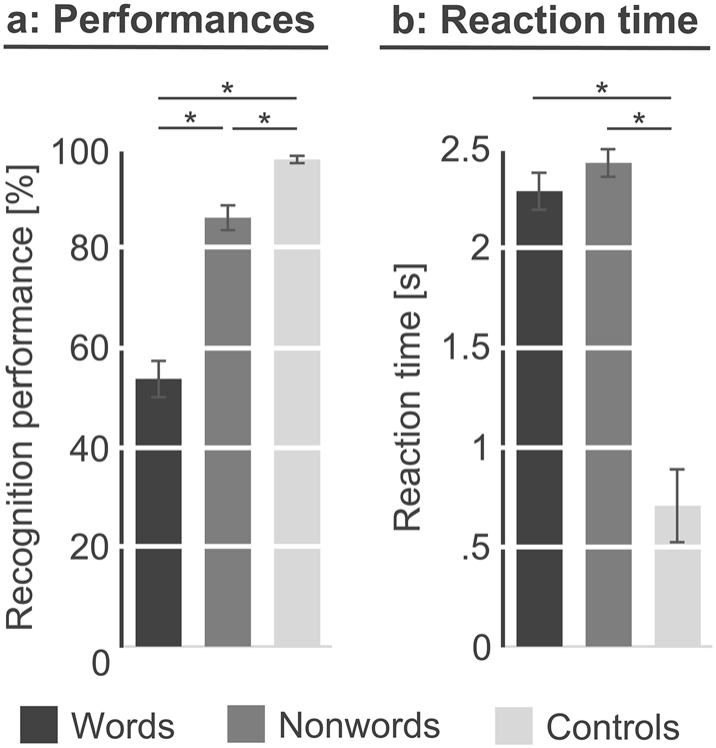
Behavioral results. Behavioral recognition performance (**a**) and reaction time (**b**) for words (dark), nonwords (medium), and controls (light). Significant differences were marked (*p < 0.001). The standard error of mean is shown as error bar

Overall, subjects correctly identified 70% of all words and nonwords presented in MC. Since recognition performances and response times were not normally distributed for most stimuli types, only non-parametric statistical tests were applied. Stimulus-specific differences in recognition performance were found (*p*_Kruskal–Wallis_ < 0.001), with the control tone (98%) being recognized more frequently than words (*p*_Mann-Whitney-U_ < 0.001; 54%) and nonwords (*p*_Mann-Whitney-U_ = 0.001, 86%; see Fig. 3a). Furthermore, nonwords were identified more often as nonwords than words were identified as words (*p*_Mann-Whitney-U_ < 0.001). In addition, differences in response times were identified (*p*_Kruskal–Wallis_ < 0.001). While the control tone (0.7 s) was recognized faster than words (*p*_Mann-Whitney-U_ < 0.001; 2.3 s.) and nonwords (*p*_Mann-Whitney-U_ < 0.001; 2.4 s), no difference was found comparing words and nonwords (*p*_Mann-Whitney-U_ = 0.428; see Fig. 3b). Furthermore, word frequency (based on a German word corpus including literature from 2000 to 2010) was correlated with recognition performance (*p*_[Spearman]_ = 0.002, r = 0.477), but missed a significant influence on reaction time (*p*_[Spearman]_ = 0.053, *r* = − 0.308).

### General linear model

**Fig. 4:**
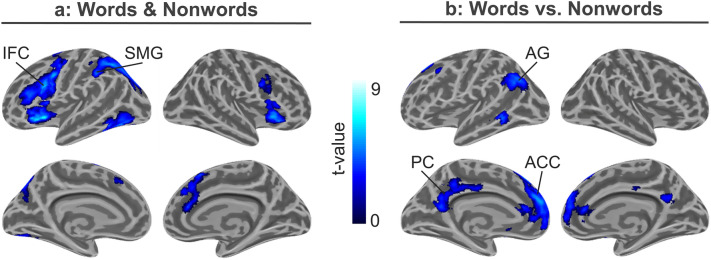
GLM results. Statistical parametric maps of cortical brain activation during non-lexical decoding of words and nonwords vs. a control tone (**a**) as well as words against nonwords (**b**)

Decoding words and nonwords elicited stronger activations compared to the control tone in left hemispheric brain regions, including the inferior frontal cortex (IFC; pars opercularis), the insular cortex and precentral cortex, the supramarginal gyrus (SMG) and superior parietal lobule, as well as the ventral occipitotemporal cortex. In addition, activation was found within the right frontal cortex (see Fig. 4a). When comparing words against nonwords (lexicality effect), enhanced activation of the left anterior cingulate cortex (ACC), the angular gyrus (AG) and precuneus (PC) were found (see Fig. 4b). Furthermore, left posterior middle temporal and subcortical activity was increased during word decoding. Vice versa, no enhanced activation for nonwords was found. For further information regarding peak activations, see Tables 1 and 2.

**Table 1:** GLM of words and nonwords

Size	Lable	L/R	*x*	*y*	*z*	*z*-value	ROI
2141	Insular cortex	L	– 30	22	– 7	> 8	–
	Inferior frontal cortex (pars opercularis)	L	– 42	8	29	6.9	#1
	Inferior frontal cortex (pars triangularis)	L	– 42	28	17	4.9	–
1406	Superior parietal lobule	L	– 22	– 72	50	5.9	–
	Superior parietal lobule	L	– 28	– 54	47	5.1	–
	Supramarginal gyrus	L	– 48	– 38	47	4.6	#2
596	Insular cortex	R	40	22	– 7	7.5	–
	Insular cortex	R	32	26	2	6.4	–
	Inferior frontal cortex (pars opercularis)	R	48	16	5	4.2	–
470	Supplementary motor area	R	2	14	53	5.6	–
	Superior frontal cortex (medial)	R	0	22	44	5.4	–
	Anterior cingulate cortex (supracallosal)	R	8	36	20	4.7	–
337	Inferior occipital cortex	L	– 44	– 68	– 13	5.9	–
	Inferior temporal cortex	L	– 52	– 54	– 13	5.1	–
	Inferior temporal cortex	L	– 46	– 46	– 16	4.6	–
155	Inferior frontal cortex (pars opercularis)	R	44	10	26	4.9	–

MNI-coordinates of peak voxels and corresponding z-values of significant clusters (initial significance level of pVoxel < 0.001, FWE-corrected on cluster-level) for shared activations during words and nonwords decoding compared to the control tone. Additionally, cluster size (in number of voxels), hemisphere (*L* left; *R* right), and ROI number (if selected) are shown

**Table 2:** GLM of words versus nonwords

Size	Lable	L/R	*x*	*y*	*z*	*z*-value	ROI
2564	Anterior cingulate cortex (pregenual)	L	− 4	48	14	6.1	#4
	Anterior cingulate cortex (pregenual)	L	– 2	48	5	6.1	–
	Superior frontal cortex (medial)	L	− 6	44	26	6	–
581	Angular gyrus	L	− 42	− 66	38	4.9	#3
	Angular gyrus	L	− 48	− 62	32	4.7	–
	Middle temporal cortex	L	− 56	− 62	23	4.1	–
429	Middle cingulate cortex	L	− 6	− 42	38	4.5	–
	Precuneus	L	− 4	− 54	14	4.3	#5
	Middle cingulate cortex	L	− 2	− 22	38	4.1	–
185	Olfactory cortex	L	− 14	6	− 13	5.2	–
	Ventral striatum	L	− 10	14	− 1	4.9	–
	Hippocampus	L	− 14	− 4	− 13	4.9	–
123	Amygdala	R	14	6	− 13	4.7	–
	Substantia nigra (pars reticulata)	R	8	− 6	− 13	4.5	–
97	Middle temporal cortex	L	− 60	− 48	− 4	4.6	–
	Middle temporal cortex	L	− 64	− 38	− 4	3.6	–

MNI-coordinates of peak voxels and corresponding *z*-values of significant clusters (initial significance level of pVoxel < 0.001, FWE-corrected on cluster-level) for different activation during words compared to nonwords decoding. Additionally, cluster size (in number of voxels), hemisphere (*L* left; *R* right), and ROI number (if selected) are shown

### Dynamic causal modeling

To identify common model features related to specific hypotheses, all 12 models were compared based on their feature families. Strong evidence was found for models that involved modulatory input of words and nonwords on the SMG (Family 1.1, posterior probability > 0.99; where higher values indicate the probability for the corresponding family or model) when being compared against models that suggest the IFC as input stage for words and nonwords (Family 1.2, posterior probability < 0.01). In addition, models involving bidirectional connections between the sublexical system and the phonological buffer (Family 2.2, posterior probability = 0.72) were favored over models with unidirectional connections (Family 2.1, posterior probability = 0.28). Furthermore, Bayesian model selection preferred models involving additional modulation while decoding meaningful words onto the connection toward the AG (Family 3.1, posterior probability = 0.80) rather than a modulation on the connection toward the ACC (Family 3.2, posterior probability = 0.14) or PC (Family 3.3, posterior probability = 0.06). For details, see Table 3.

**Table 3:** DCM model comparison

	a: Difference in log-evidence					b: Posterior probability				
	SMG → IFC		IFC → SMG		Family 3	SMG → IFC		IFC → SMG		Family 3
	↑	↑↓	↑	↑↓		↑	↑↓	↑	↑↓	
AG	32.0	33.6	2.0	3.4	8.5	0.134	0.666	< 0.001	< 0.001	0.800
ACC	32.0	29.3	0.0	1.1	0.0	0.134	0.009	< 0.001	< 0.001	0.140
PCC	29.7	30.9	0.9	3.1	2.1	0.013	0.043	< 0.001	< 0.001	0.060
Family 2	0.0	4.8	0.0	4.8		0.280	0.720	0.280	0.720	
Family 1	176.9		0.0			> 0.999		< 0.001		

Difference in log-evidence comparing individual models against the worst model (a). For model families, log-evidences were first summed across each family before the difference was calculated compared to the worst family. A difference in log-evidence of “0” represents the worst model or family. Posterior probabilities for individual models and model families (b), where higher values indicate the probability for the corresponding model or family

^↑^Unidirectional connection (bottom-up)

^↑↓^Bidirectional connection (bottom-up and top-down)

**Fig. 5:**
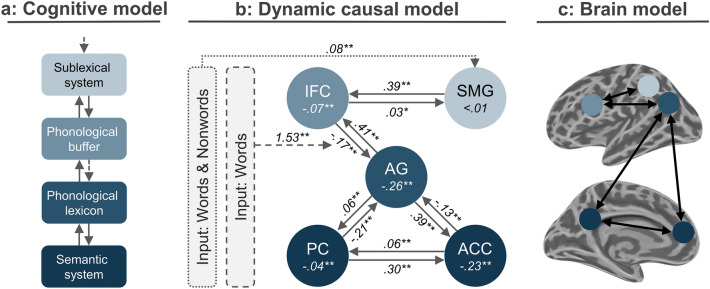
DCM results. Cognitive model for non-lexical decoding (**a**) with connectivity strengths of the most likely DCM model (**b**) and resulting brain mapping (**c**; ROIs shown as circles). Connectivities are shown within each circle (self-connectivity; A matrix), as continuous lines (static connectivity; A matrix), as dotted lines (modulatory experimental influences; C matrix), or as stitched lines (experimental influences on the effective connectivity; B matrix). Significant connections were marked (*posterior probability > 0.95; **posterior probability > 0.99)

As expected from the family comparison, the most likely model (posterior probability = 0.66, difference in log-evidence: 33.6) involved modulatory input of words and nonwords to left supramarginal activity, which connected to the IFC in a bidirectional manner (see Fig. 5b). Furthermore, words modulated the connectivity from IFC to AG. In this model, most connections (except for intrinsic SMG connection, posterior probability = 0.5) as well as all modulatory inputs were significant (posterior probability > 0.95). For details, see Fig. 5.

## Discussion

In the present study, we used Morse code (MC) and fMRI to probe the non-lexical route in reading followed by a lexical decision. Common brain activations related to non-lexical decoding of words and nonwords were found within the left SMG and adjacent superior parietal lobule, as well as the left IFC, while meaningful words additionally recruited the AG, ACC, and PC. We employed DCM to further disentangle the interaction of activated brain regions. This way, we tested various models of non-lexical decoding and word comprehension taking into account the DRC and CDP models for reading, attributing specific functions to the SMG, the IFC, and the AG (see Fig. 5c).

The winning DCM model that explains the measured BOLD signal best suggests modulation of SMG activity by the word and nonword condition. The SMG interacts with the opercular part of the IFC via bidirectional (bottom-up and top-down) connections. Furthermore, the word condition additionally modulated the connection from the IFC (pars opercularis) to the AG. The AG was then connected with both ACC and PC. Our analyses provide evidence that the SMG, rather than the IFC, serves as an input channel to the non-lexical decoding network, supporting the hypothesis of left SMG involvement in sublexical letter conversions (system) and left opercular part of the IFC involvement in phonological working memory (buffer; Family 1.1). Information flow, as suggested by DCM, proceeds in a bidirectional manner between the SMG and IFC (Family 2.2). To enable comprehension of meaningful words, the IFC connects to the left AG (only in the word condition), suggesting an involvement in the phonological lexicon (Family 3.1). Finally, the AG interacts with the ACC and PC, presumably reflecting the access to semantic memory representations. As such, our analyses support the notion that the left SMG and IFC (pars opercularis) host components of the non-lexical decoding network, i.e., grapheme to phoneme conversion and phoneme assembly, respectively. Furthermore, the AG seems to serve as a bidirectional interface between the non-lexical decoding network and the semantic network, accessed when a word is identified as such.

At the behavioral level, the lack of difference in RT between words and nonwords suggests that no lexical decoding strategy was applied. At the same time, meaningless nonwords were identified more frequently than meaningful words. Increased recognition performance for nonwords can be explained by potential translation errors during decoding. The combination of the three letters learned in the current experiment can yield 89 German words and 1639 nonwords. Therefore, a translation error leads to an 18.4 times higher chance of transforming a word into a nonword (than vice versa), explaining how differences in recognition performance between both stimuli types might have originated. In addition, word recognition performances were correlated with frequencies, showing that more frequently used words were recognized more often than less frequent words. A relationship between word frequency and decoding behavior is often observed in reading and can be related to phonological, lexical or semantic stimulus representations (Graves et al. 2010). However, the lack of difference in RT between words and nonwords suggests that no accelerated lexical decoding strategy was applied. Hence, decoding meaningful words benefits from more robust phonological and semantic memory representations, allowing a more reliable stimulus identification (Desai et al. 2020). For a more detailed discussion of MC decoding behavior, see Junker et al. 2020.

Decoding words and nonwords elicited brain activations and changes in effective connectivity within and between the SMG and IFC, brain regions typically associated with language decoding. Studies using different stimulus types (DeMarco et al. 2017), linguistic properties (Graves et al. 2010; Protopapas et al. 2016), or orthographies (Mei et al. 2014; Rueckl et al. 2015) provide strong evidence, that both regions are critically involved in non-lexical decoding. However, since the sublexical system and phonological buffer are equally affected by these factors, the exact role of the IFC and SMG during decoding remains to be fully elucidated. Letter-selective activations have been found within the left SMG (Joseph et al. 2003, 2006), suggesting a specific role of the SMG in the sublexical system. Furthermore, anodal transcranial direct-current stimulation over the left inferior parietal lobule (including the SMG) interferes with the acquisition and maintenance of novel grapheme-phoneme mappings, which is heavily based on the performance of the sublexical system (Younger and Booth 2018). Repetitive transcranial magnetic stimulation (rTMS) over the left pars opercularis of the IFC impairs phonological working memory (Nixon et al. 2004). By contrast, rTMS over the pars triangularis interferes with semantic processing (Whitney et al. 2011) rather than phonological working memory (Nixon et al. 2004). This dissociation of posterior (BA44, precentral) and anterior frontal brain regions (BA45) involved in phonological and semantic processing, respectively, is further supported by multiple studies investigating brain function (Liakakis et al. 2011) and effective connectivity (Heim et al. 2009). During non-lexical decoding, stronger effective connectivity toward posterior frontal regions can be observed. Vice versa, lexical decoding of irregular words led to enhanced effective connectivity toward anterior frontal regions (Mechelli et al. 2005), a process that is supported by semantics (Boukrina and Graves 2013). Although these studies demonstrated direct connectivity from the fusiform gyrus to the IFC, their results still support a functional segregation within the IFC. Interestingly, our DCM analysis favored models including additional top-down connections between IFC (pars opercularis) and SMG, a connection found to be positively correlated with reading skills in healthy children that more strongly rely on non-lexical decoding (Cao et al. 2008). This bidirectional information flow might be enabled via the third branch of the superior longitudinal fasciculus (Frey et al. 2008), allowing for fast communication between the sublexical system (SMG) and phonological buffer (opercular part of the IFC).

Furthermore, activations during word as compared to nonword decoding were found in the left AG, ACC, and PC, key regions of the DMN. The DMN is one of the most robust resting-state networks that is associated with higher cognitive functions, such as episodic and semantic memory, prospection, and theory of mind (Spreng and Grady 2009). During periods of rest, the regions of the DMN are functionally and effectively connected, with the AG serving a driving role (Sharaev et al. 2016). In contrast, the DMN is deactivated during tasks (task-negative network), presumably to suppress internal thoughts (Barber et al. 2017) and to guide goal-directed behavior (Daselaar et al. 2009). This deactivation, however, is reduced during semantic processing of e.g., written words, indicating an involvement of the DMN in representing semantic rather than perceptual or phonological stimulus features (Wirth et al. 2011). Accordingly, brain regions of the DMN are more strongly engaged while processing meaningful words compared to meaningless nonwords (Lin et al. 2016), independent of the phonology and orthography of a language (Dehghani et al. 2017). In addition to semantics, the DMN is involved in the formation of episodic memory (Baldassano et al. 2017). Episodic memory is enhanced by the semantic richness of an event (Craik and Lockhart 1972) and therefore intertwined with semantic memory (for review, see Renoult et al. 2019). Overall, the here identified comprehension network overlaps extensively with the DMN, a brain network that hosts semantic memory functions required to process highly abstract features that are independent of the sensory modality (Xu et al. 2017).

Our DCM analysis suggests a distinct role of the AG, possibly serving as a bidirectional interface between phonological working memory (IFC) and the semantic system (as accessed through the phonological lexicon). Activations within the AG seem to be driven by both phonological and semantic features (Kim 2016). Interestingly, Barbeau et al. (2017) found a correlation between AG activation and reading speed of a newly acquired language suggesting that enhanced phonological representations facilitate word recognition during non-lexical decoding. At the same time, the left AG represents one of the most reliably activated brain regions in imaging studies on the neural correlates of the semantic system (Binder et al. 2009), and some authors have even suggested functional subdivisions of the AG involved in searching (dorsal) and mapping (medial/ventral) of semantic representations (Seghier et al. 2010). The parallel processing of phonological and semantic features within the AG is further supported by Taylor et al. (2013), suggesting an involvement in the phonological lexicon as well as the semantic system. In this way, the left AG might link assembled phonology to prior knowledge via phonological representations, making it important for reading acquisition, especially in the early phase when non-lexical decoding is still the leading strategy. As orthographic experience increases over time, processing shifts from non-lexical toward lexical decoding, allowing a direct mapping of word orthography onto semantic representations, bypassing phonology. This reduction in non-lexical decoding goes along with reduced activity of the AG (Seghier 2013), although the AG maintains a driving role at least during periods of rest (Sharaev et al. 2016). However, how exactly orthographic and semantic representations interact during lexical decoding remains to be fully elucidated.

Although the effects found here are consistent with literature examining language decoding and word comprehension, the results represent only part of the story. First, the models used here include only those aspects that are specific to language decoding. Other aspects, such as attention, cognitive control, and error monitoring are not part of the model but are important for decoding as well. Accordingly, brain activities that are not specific to language decoding are also to be expected. Second, we identified additional brain regions involved in MC decoding (insular and ventral occipitotemporal cortex) and comprehension (posterior middle temporal cortex). Although the involvement of the insular cortex in various linguistic processes was found (Borowsky et al. 2006), it is still under debate whether the insular cortex plays a crucial role in decoding since patients with insula lesions usually recover from initial reading deficits quickly (within weeks or months) (Uddin et al. 2017). Furthermore, although the ventral occipitotemporal cortex is associated with the ventral visual stream and visual language decoding (e.g., Lerma-Usabiaga et al. 2018; Taylor et al. 2019), studies in blind persons show recruitment of the ventral occipitotemporal cortex also while decoding tactile Braille (Dzięgiel-Fivet et al. 2021). In addition, we identified stronger engagement of the posterior middle temporal cortex (MTC) while decoding words compared to nonwords, but did not include this region in our DCM analysis. The MTC represents one core region of the semantic system (Binder et al. 2009), which is involved in the processing of spoken and written language (Rueckl et al. 2015), as well as in the processing of gestures (Papeo et al. 2019). In general, the MTC can be divided into anterior and posterior subregions, involved in semantic representation and control, respectively (Jackson 2021). Disruption of left posterior MTC activity leads to an impairment of demanding semantic associations (e.g., salt—grain), but not of automatic semantic associations (salt-pepper) or non-semantic controls (Whitney et al. 2011). Thus, the left posterior MTC (as identified here) plays a crucial role in semantic control, but may not be central to semantic representation. Therefore, none of these brain regions were included in the DCM analysis to keep the analysis comparable to the cognitive model for language decoding. Third, we interpreted the effects observed here only with regard to the cognitive model of reading. For instance, the AG is also involved in a variety of tasks that involves the processing and manipulation of concepts, including the semantic processing of words (Rueckl et al. 2015) and sentences (Ettinger-Veenstra et al. 2016). Hence, the AG resembles a heteromodal hub involved in integrating semantics across various sensory modalities (for review, see Seghier 2013), rather than just an interface between the non-lexical reading network and the semantic system. In addition, besides evidence for semantic processes (Kozlovskiy et al. 2012; Zhao et al. 2017), the ACC is often associated with domain-general processes such as cognitive control (Blanco-Elorrieta and Pylkkänen 2016), where stronger activations can be observed during demanding conditions (Aben et al. 2020). Increased cognitive control is also necessary when processing meaningful words, as these require more elaborate and demanding processing compared to meaningless nonwords due to their additional phonological and semantic representations. However, the study design did not allow us to distinguish semantic processes from domain-general processes such as demanding cognitive control.

## Limitations

Some limitations regarding the decoding behavior and stimulus material must be mentioned. Overall, our sample revealed a large variance in recognition performance, especially for words. While some subjects learned the Morse code very well (high-performers, word performance ≥ 55%), the learning success of others is questionable (low-performers, word performance < 55%). When comparing high- and low-performers, however, no differences in eigenvariates while decoding words can be found, neither in the IFC (*p* > 1), SMG (*p* > 1), AG (*p* = 0.254), PC (*p* > 1) nor ACC (*p* > 1; see Supplementary Fig. 1). Therefore, we decided to analyze all subjects together, regardless of their word performance in the lexical-decision task. However, future studies should establish an additional learning criterion after the completion of all audiobook lessons, which must be achieved by the subjects to be included in the final study population. In this way, further effects could be observed, which might have been missed here due to the heterogeneous population. In addition, future studies might use a more homogeneous set of words in which, for example, words with low frequencies are avoided. This selection would also have a positive influence on the performance in the lexical-decision task, since especially low frequency words were identified less often by the subjects. However, since we aimed to minimize the amount of content to be learned (only 12 different letters) as well as the involvement of working memory while decoding (only stimuli consisting of 3 letters), we had to rely on a heterogeneous stimulus set here. Furthermore, the nonwords used here contained 10 unpronounceable nonwords and 30 pronounceable pseudowords. Since unpronounceable nonwords could already be identified earlier due to their usage of illegal GPC, complete non-lexical processing cannot be guaranteed for those stimuli. However, the influence on the results is to be considered small, as no faster processing speed was found for real nonwords (2.5 s) compared to regular pseudowords (2.3 s; p_Mann-Whitney-U_ = 0.082).

## Conclusion

In this study, DCM was used to map conceptually adapted computational models for reading (DRC, CDP) onto brain regions showing robust activations during non-lexical decoding and word comprehension of MC. We considered interactions between five different brain regions and suggested an involvement in the serial letter conversion (SMG), phonological working memory (opercular part of the IFC) as well as in representing phonological (AG) and semantic features of known words (AG, ACC, PC). Overall, the AG seems to play a specific role, as it enables the interaction between the non-lexical decoding network (SMG, IFC) and the semantic memory system. As such, the AG is likely to host phonological and semantic representations and to serve as a bidirectional interface between the external (task-positive network) and the internal world (task-negative network).

### Supplementary Information

Below is the link to the electronic supplementary material.

Supplementary file1 (DOCX 153 KB)

## Data Availability

The data that support the findings of this study are available from the corresponding author upon reasonable request.

## Abbreviations

ACC: Anterior cingulate cortex
AG: Angular gyrus
BA: Brodmann area
CDP: Connectionist dual-process
DCM: Dynamic causal modeling
DMN: Default mode network
DRC: Dual-route cascade
fMRI: Functional Magnetic Resonance Imaging
GLM: General linear model
GPC: Grapheme-phoneme correspondence
IFC: Inferior frontal cortex
MC: Morse code
MNI: Montreal Neurological Institute
MTC: Middle temporal cortex
PC: Precuneus
ROI: Region of interest
RT: Reaction time
SD: Standard deviation
SMG: Supramarginal gyrus
SPM: Statistical Parametric Mapping
