# Supplementary material for: The angular gyrus serves as an interface between the non-lexical reading network and the semantic system: evidence from dynamic causal modeling
Source: Brain Struct Funct. 2023 Mar 11;229(3):561–75. doi: 10.1007/s00429-023-02624-z (PMC10978681; doi:10.1007/s00429-023-02624-z)
Supplement: Supplementary file 1 — Supplementary file1 (DOCX 153 KB) [file 429_2023_2624_MOESM1_ESM.docx]

**Supplementary**

**Supplementary Table 1 – List of stimuli**

List of all MC stimuli used for training (only letters) and the subsequent lexical-decision task. Additionally, the word frequency based on the DWDS core corpus 21 (literature from 2000-2010, comprising 121 million words) is shown as instances per million words.

| Training |  | Lexical-decision task | | | | | | |
| --- | --- | --- | --- | --- | --- | --- | --- | --- |
| Letters |  | Words (with frequency) | | | |  | Nonwords | |
|  |  |  |  |  |  |  |  |  |
| A |  | AMT | 6.15 | NEU | 198 |  | ADN | NED |
| D |  | ARM | 8.16 | NOT | 4.05 |  | DRU | NER |
| E |  | AST | 1.16 | ODE | 0.13 |  | DSA | NIG |
| G |  | AUE | 0.12 | OMA | 0.95 |  | DUS | ODA |
| I |  | DEO | 0.14 | ORT | 16.0 |  | ENS | ODN |
| M |  | DOM | 0.95 | OST | 1.15 |  | GEM | OGS |
| N |  | DUO | 0.22 | RAD | 2.03 |  | GID | ONS |
| O |  | EGO | 0.32 | ROT | 15.6 |  | GIU | RAI |
| R |  | EID | 0.40 | RUM | 0.60 |  | GMI | RIR |
| S |  | EIS | 2.27 | SAU | 0.46 |  | GNI | RUO |
| T |  | EMU | 0.05 | SEE | 3.94 |  | IDI | SEG |
| U |  | ENG | 11.0 | SET | 1.74 |  | IRM | SIA |
|  |  | GAG | 0.21 | SOG | 0.40 |  | ISG | SNU |
|  |  | GAS | 1.14 | SUD | 0.05 |  | MAT | SRU |
|  |  | GEN | 1.45 | TAG | 66.2 |  | MES | TIA |
|  |  | GIN | 0.24 | TAU | 0.26 |  | MIM | TIS |
|  |  | GNU | 0.77 | TEE | 2.55 |  | MNI | TRO |
|  |  | GUT | 198 | TOD | 14.1 |  | MRA | TUG |
|  |  | MAI | 12.2 | TON | 10.6 |  | MUG | UDI |
|  |  | MUT | 3.18 | TOR | 3.57 |  | NAR | UMN |

**Supplementary Table 2 – Subject-specific behavior**

Behavioral recognition performances (mean only) and reaction times (mean and standard deviation (SD)) for each subject and condition (words, nonwords, controls).

|  |  | Recognition performance | | | | |  | Reaction time | | | | | | | |
| --- | --- | --- | --- | --- | --- | --- | --- | --- | --- | --- | --- | --- | --- | --- | --- |
| ID |  | Words |  | Nonwords |  | Controls |  | Words | |  | Nonwords | |  | Controls | |
|  |  | mean |  | mean |  | mean |  | mean | SD |  | mean | SD |  | mean | SD |
|  |  |  |  |  |  |  |  |  |  |  |  |  |  |  |  |
| 1 |  | 63 |  | 80 |  | 92 |  | 2.7 | 1.06 |  | 2.9 | 0.88 |  | 0.9 | 0.56 |
| 2 |  | 33 |  | 85 |  | 100 |  | 2.3 | 0.86 |  | 2.4 | 1.27 |  | 1.2 | 0.12 |
| 3 |  | 80 |  | 93 |  | 100 |  | 1.8 | 0.78 |  | 2.3 | 0.77 |  | -1.9 | 0.18 |
| 4 |  | 63 |  | 73 |  | 96 |  | 2.4 | 0.72 |  | 2.6 | 1.86 |  | -1.7 | 0.28 |
| 5 |  | 63 |  | 90 |  | 100 |  | 2.4 | 1.08 |  | 2.6 | 0.81 |  | -0.8 | 0.71 |
| 6 |  | 65 |  | 95 |  | 100 |  | 1.8 | 0.67 |  | 2.2 | 0.69 |  | 1.0 | 0.07 |
| 7 |  | 73 |  | 100 |  | 100 |  | 1.9 | 0.61 |  | 2.0 | 0.76 |  | 1.0 | 0.45 |
| 8 |  | 50 |  | 88 |  | 100 |  | 2.3 | 0.91 |  | 2.8 | 0.85 |  | 1.1 | 0.15 |
| 9 |  | 70 |  | 100 |  | 100 |  | 1.7 | 0.78 |  | 2.6 | 0.92 |  | 1.0 | 0.21 |
| 10 |  | 30 |  | 93 |  | 100 |  | 2.4 | 0.59 |  | 2.8 | 0.91 |  | 1.4 | 0.44 |
| 11 |  | 40 |  | 93 |  | 92 |  | 2.2 | 0.93 |  | 2.3 | 1.07 |  | 0.9 | 0.28 |
| 12 |  | 60 |  | 88 |  | 100 |  | 1.7 | 0.63 |  | 2.6 | 1.32 |  | 1.2 | 0.22 |
| 13 |  | 85 |  | 98 |  | 100 |  | 2.0 | 1.03 |  | 2.7 | 0.77 |  | 1.1 | 0.14 |
| 14 |  | 95 |  | 100 |  | 100 |  | 1.9 | 0.77 |  | 2.0 | 0.66 |  | 1.0 | 0.38 |
| 15 |  | 68 |  | 95 |  | 100 |  | 1.9 | 1.87 |  | 2.6 | 1.55 |  | 1.0 | 0.10 |
| 16 |  | 51 |  | 50 |  | 81 |  | 2.5 | 1.03 |  | 2.4 | 0.87 |  | 1.1 | 0.12 |
| 17 |  | 45 |  | 90 |  | 92 |  | 2.3 | 0.89 |  | 2.6 | 1.02 |  | 1.5 | 0.63 |
| 18 |  | 48 |  | 93 |  | 100 |  | 2.1 | 0.81 |  | 2.2 | 0.56 |  | 1.3 | 0.19 |
| 19 |  | 38 |  | 78 |  | 100 |  | 1.8 | 0.79 |  | 2.1 | 0.72 |  | 1.2 | 0.36 |
| 20 |  | 40 |  | 58 |  | 92 |  | 1.8 | 0.64 |  | 1.7 | 0.71 |  | -1.2 | 0.98 |
| 21 |  | 78 |  | 95 |  | 100 |  | 2.1 | 0.68 |  | 2.5 | 0.65 |  | 1.6 | 0.23 |
| 22 |  | 55 |  | 53 |  | 100 |  | 2.1 | 0.65 |  | 2.2 | 0.72 |  | 1.5 | 0.35 |
| 23 |  | 5 |  | 85 |  | 100 |  | 4.5 | 2.55 |  | 1.1 | 0.89 |  | 1.1 | 0.18 |
| 24 |  | 40 |  | 95 |  | 96 |  | 1.9 | 0.89 |  | 2.2 | 0.71 |  | 1.3 | 0.47 |
| 25 |  | 33 |  | 95 |  | 100 |  | 3.0 | 0.93 |  | 2.9 | 0.97 |  | -1.9 | 0.31 |
| 26 |  | 80 |  | 98 |  | 100 |  | 2.3 | 1.07 |  | 2.6 | 1.00 |  | 1.3 | 0.15 |
| 27 |  | 70 |  | 93 |  | 100 |  | 2.4 | 1.41 |  | 2.7 | 0.78 |  | 1.4 | 0.21 |
| 28 |  | 65 |  | 95 |  | 96 |  | 2.4 | 0.84 |  | 2.7 | 1.04 |  | 1.3 | 0.44 |
| 29 |  | 50 |  | 90 |  | 100 |  | 2.7 | 1.04 |  | 2.8 | 1.91 |  | 1.0 | 0.32 |
| 30 |  | 18 |  | 93 |  | 100 |  | 2.8 | 0.89 |  | 2.6 | 0.39 |  | 1.2 | 0.34 |
| 31 |  | 35 |  | 80 |  | 96 |  | 2.7 | 0.65 |  | 2.7 | 0.72 |  | 1.1 | 0.54 |
| 32 |  | 63 |  | 80 |  | 92 |  | 2.6 | 1.70 |  | 2.7 | 1.30 |  | -1.1 | 0.92 |
| 33 |  | 25 |  | 65 |  | 100 |  | 1.9 | 0.60 |  | 1.8 | 0.65 |  | 1.4 | 0.25 |
|  |  |  |  |  |  |  |  |  |  |  |  |  |  |  |  |
| mean |  | 54 |  | 86 |  | 98 |  | 2.3 | 0.9 |  | 2.4 | 0.9 |  | 0.7 | 0.3 |


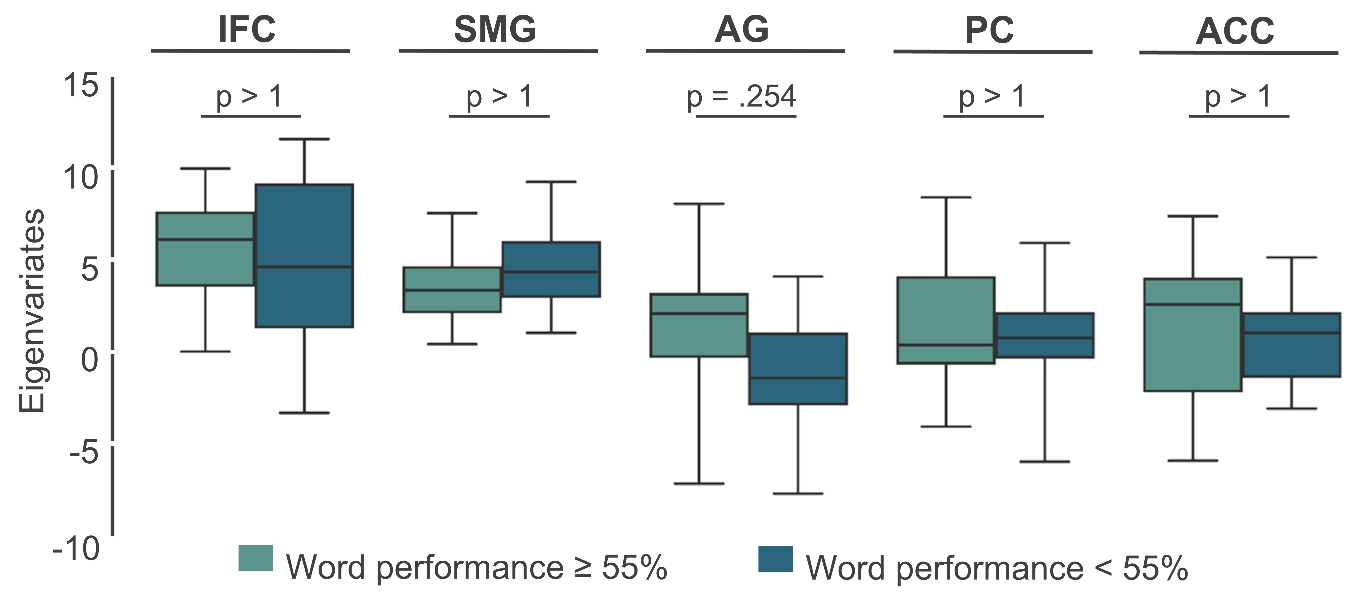


**Supplementary Figure 1 – Eigenvariates**

Regional eigenvariates while decoding words. Dependent on their word decoding performance, participants were labeled either as high- or low-performers before their corresponding eigenvariates were compared using a t-test. All results were Bonferroni corrected for multiple comparisons.
